# Social construction of the experience of living with chronic kidney disease^1^

**DOI:** 10.1590/1518-8345.2439.3028

**Published:** 2018-08-09

**Authors:** Claudia Andrea Ramírez-Perdomo, Mari Carmen Solano-Ruíz

**Affiliations:** 2 Doctoral student, Facultad de Enfermería, Universidad de Antioquia, Medellín, Ant, Colombia. Associate Professor, Departamento de Enfermería, Universidad Surcolombiana, Neiva, Huila, Colombia.; Universidad Surcolombiana, Departamento de Enfermería, Universidad Surcolombiana, Neiva, Huila, Colombia; 3 PhD, Full Professor, Departamento de Enfermería, Universidad de Alicante, Alicante, Spain.

**Keywords:** Kidney Disease Chronic, Kidney Transplantation, Qualitative Research, Hermeneutic, Nursing, Care

## Abstract

**Objective:**

To understand the experience of people living with Chronic Kidney Disease who have been transplanted, from the meanings constructed based on the experienced phenomenon.

**Method:**

Hermeneutic-phenomenological study based on the five lifeworld existentials, according to Van Manen’s theoretical framework. Eleven transplanted patients participated in the study and data collection was carried out through semi-structured interviews, after approval of the study by the Ethics Committee of the University of Antioquia.

**Results:**

The theme of Living with Chronic Kidney Disease emerged, and the subthemes were grouped as lifeworld existentials of Temporality: something unexpected, being present and not seeing it, being young and sick. Relationality: support, feeling stuck and Terminal Chronic Renal Failure. Spatiality: changes in life, sadness and depression. Corporeality: body deterioration and changes in sex life. Materiality: effects on the economic status.

**Conclusions:**

The care provided to people must be oriented in order to recognize their individualities, understanding what the illness means for the individual and his family, how they live with it and what the changes are, leading them to modify their lives and start a long process, such as living with a chronic disease.

## Introduction

The main challenges faced by health systems are aging and chronic diseases. Among these, Chronic Kidney Disease is characterized by being a debilitating disease, caused by a gradual and progressive loss of renal function, which affects the person and his surroundings^1^. The number of people with Chronic Kidney Disease and Terminal Chronic Renal Failure continues to increase exponentially, being a public health problem that could reach severe epidemic proportions. There are several important factors for its development, such as aging, cardiovascular diseases and type II diabetes mellitus, considered to be responsible for its increasing incidence^2^.

In 2010, more than 2 million people in the world were treated for this disease^3^. In the United States, during the year 2013, the incidence of people diagnosed with this disease varied from 6.3 to 9.2%^4^. It is estimated that, by 2030, approximately 2.2 million people will require renal replacement therapy^5^. The main treatment options are: kidney transplantation or dialysis, and given the limitations of kidney transplantation, it is recognized that most people enter a renal replacement therapy^2^.

The disease diagnosis implies a readjustment in the different spheres of the persons’ life, leading them to face multiple changes in their physical, psychological and affective conditions, life system, and family and work relationships, as well as in their surroundings, which affect their lives.

Several studies have addressed this phenomenon, including diet issues and changes in protein-calorie intake, which cause an increase in malnutrition during the initial stage of renal replacement therapies, leading to increased morbidity and mortality in these people^6^. They also address changes in the patient’s sex life due to hormonal changes, symptoms associated with the disease and dialysis treatment^7^ and the importance of decision making in relation to treatment modality^8^. 

Other aspects related to the effects on body image and to treatment, considered as a mechanism for surviving the disease^9^, and the difficulties regarding adherence to treatment^10^, such as experiences and religious practices contribute to decision making. The coping strategies used to assume the disease and dialysis^11^, as well as the coping strategies used to assume the treatment and keep trying to live despite the disease^1^ ^,^ ^12^, are important elements that involve changes in the patient’s life. 

Based on the ideas presented, in Nursing, it is important to care for the individual with Chronic Kidney Disease by applying a holistic approach built from the social dimension, in order to provide a discipline with an experiential and conceptual basis aiming at strengthening the professional practice, considering the experiences of people affected by the disease as its key aspects.

In this context, the objective of this study was to understand the experience of people living with Chronic Renal Disease who have been transplanted, from the meanings constructed based on the experienced phenomenon.

## Method

The hermeneutic-phenomenological qualitative research method proposed by Van Manen was adopted, aiming, through face-to-face interviews, to reconstruct the meaning of the experience of people living with Chronic Renal Disease. The socio-personal environment was explored with the aim to identify key aspects that allow a deep understanding of their experience, and derive from the knowledge obtained, a set of strategies that allow improving the care provided by the nursing staff to people with this health condition.

The analysis was centered on the lifeworld existentials^13^: Relationality, it identifies the relationships that people maintain in their shared space, and leads to the reflection about how relationships can influence the perception of the phenomenon, recognizing common features in the other, the social components that give meaning to life^13^. Corporeality, it is the immersion in the corporeal world, things are consciously or unconsciously revealed or hidden in the encounters with other people, the body language changes during these encounters, it allows to identify how the body experiences, feels, perceives and manages the phenomenon^13^. Spatiality, it is interpreted as having Cartesian properties with universal coordinates and distances, and it is interested is in how space is felt and gives meaning to the phenomenon^13^, “the spatiality of man is something inseparable from his corporeal being … “grounded” in his corporeality”^14^. Temporality, it is examined in relation to how time is experienced, and is correlated with a subjective time, the experience meanings are interpreted at a particular time^13^. Materiality, it concerns on how things are experienced^13^, and describes how “intramundane or useful bodies”^14^ ^)^ assign a meaning.

Study sample consisted of 11 participants with Chronic Renal Disease, who have been transplanted and were residents in the city of Neiva, Colombia. They were selected by a random^15^ non-probabilistic criterion-oriented sampling, from the database provided by the Surcolombiana Transplantation Unit, composed of 81 transplant recipients. The inclusion criteria for data collection were: be over 18 years of age, having received kidney transplantation for over 6 months and be resident in Neiva. A pseudonym was immediately assigned to the participant’s data, and it was decided to identify them by the initials of their names. 

Information was collected from December 2015 to January 2017. A person was initially contacted and the process of linking the participants was continued based on that interview. All the contacted people agreed to collaborate and the authors had no bond with these people. In-depth interviews were used as technique for collecting the information. The researcher contacted the participants by telephone and interviewed them at their residences, starting from the following general question: Can you tell me about your experience since you were diagnosed with Chronic Kidney Disease? Additionally, a list of questions was elaborated that allowed to deepen on the phenomenon. The interviews lasted one hour to one hour and thirty minutes and were recorded, listened and transcribed in full by the researcher.

The Ethics Committee of the University of Antioquia approved the study, Document # CEI-FE2015-05. The Resolution 8430 of 1993 and the ethical principles of beneficence, autonomy, privacy, freedom of expression and sentiments were considered.

The analysis was carried out according to Van Manen’s method: reading and re-reading the interviews, searching and understanding the etymological roots, searching for emerging themes, lighting and illustration on the phenomenon, searching for common patterns, writing and re-writing centered on the lifeworld existentials^13^. 

The following criteria were considered^16^: credibility and possibility of obtaining confirmation were achieved through textual transcription of the interviews by the researcher, and the results were reviewed by qualitative research experts and returned to the participants. Regarding transferability, it is expected that the results of this study serve to find similarities or divergences with the findings of other studies addressing the phenomenon.

## Results

**Figure 1:**
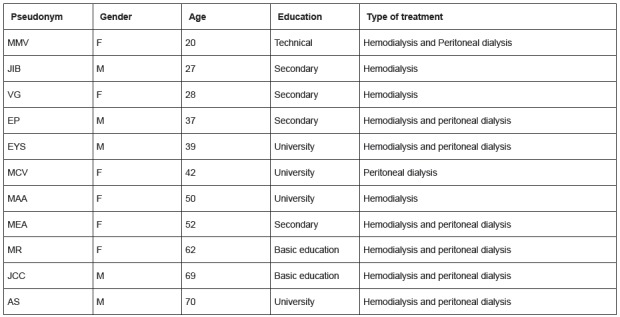
Characterization of the study participants, Neiva, Huila, Colombia 2015-2017

Figure 1 presents the characterization of the participants residents in Neiva, 5 men and 6 women; minimum age of 20 years, maximum of 70 years, 6 received Peritoneal Dialysis and Hemodialysis, 4 Hemodialysis and 1 Peritoneal Dialysis.

Three themes emerged: Living with Chronic Kidney Disease, Being under treatment, and Changes in life after transplantation. This article addresses the descriptions of the theme of Living with Chronic Kidney Disease, and the discussion subthemes presented here were considered from the perspective of the lifeworld existentials^13^.

**Figure 2:**
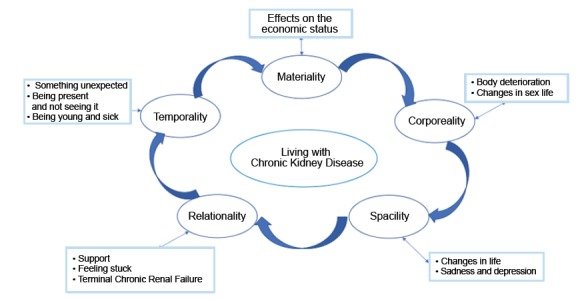
Relationship between the lifeworld existentials and sub-themes

Figure 2 shows the relationship between the lifeworld existentials and the sub-themes, allowing to recognize that experience is a relationships network and not a series of events that take place during the life of human beings, which is consistent with the hermeneutical phenomenology. Next, these are described and illustrated with sections of the interviews.

Temporality meaning for the participants: something unexpected, being present and not seeing it, being young and sick.

Something unexpected “chronic kidney disease: a surprise”. They describe that the disease manifests as a breakdown of everyday life. The disease appears at a moment’s notice and the affected persons experience symptoms such as vomiting, adynamia, diarrhea and edema; and they visit a doctor’s office or an emergency room without even image that they have a chronic kidney disease, characterized by failure of kidney function. 

*It all started when I began to exhibit symptoms ... I began to feel sick, I had vomiting, diarrhea, a foul taste in my mouth, cramps, discouragement, initially, everything I ate I vomited, I thought I had hepatitis, anemia or some viral disease, I never imagined that I had a Chronic Kidney Disease. (EP)*

*It was 10 years ago, when I was pregnant, when my daughter was born I had edemas, I was hospitalized and a few days later they told me that I had Chronic Kidney Disease. (VG)*

Finding out that they have Chronic Kidney Disease becomes a traumatic moment, which leads them to “disbelieve” in what is happening, because during their lives, they had been considered healthy people, and thus, this situation becomes an unknown experience. They consider this process as a hard and difficult situation to deal with, also described as “for life”.

*I had never undergone any surgery or suffered a fracture, so having to lead a difficult life and becoming aware of it at a moment´s notice was a new, strange, peculiar experience to me, of course, something I had never imagined. I said: God, what is it all about? Why all this madness? Why did I get sick? and, When did this happen? I said “I cannot believe it” one wonders: Why me? (MCV)*

Being present and not seeing it. CKD becomes a battle against time. The disease announces its presence by sending alerts that are not detected early; therefore, the diagnosis is delayed for when the disease has been definitively established in the person.

*That was hard, I was born with only one kidney and I just realized that at the age of 50... they performed examinations and found that I was born with only one kidney and I was working like a toad, slow, slow and that was hard .... I was arriving late .... That was how my battle began. It has been hard. (MR)*

This problem has been present for a long time and warnings are presented, but they are not able to detect the warning signs. The presence of associated diseases as possible triggerings causes of this one are unknown and, only when they are evaluated by the doctor or undergo laboratory tests and receive the diagnosis they try to establish the association between symptoms and the disease. Participants became aware of these symptoms after the diagnosis, but due to lack of knowledge, they were unable to identify them. 

*I realized that the warnings were on, but I never paid attention. At that time, I remember that I drank alcohol on weekends and I did not feel like eating, I felt nauseous, I drank but I was not feeling well. “What’s the matter?”, “I do not feel like drinking, I do not know what’s wrong with me, I have been feeling sick”, “I realized that this happened to me every now and then”, but I did not notice anything else besides that. (EYC)*

*I was asymptomatic, I never had fluid retention, they prescribed me a paste of enalapril to control the pressure and I kept working ... I had never had fluid retention, but this time I was hospitalized with 53 kilos and at some point I was weighing 60 kilos ... I had 7 kilos of liquids. (MAA)*

Being young and sick. The young participants recognize age as a positive factor, it means that they have a lot of time to live, on the other hand, it is perceived as a factor that makes it difficult to take on their daily activities. All this associated with the fact of not being able to perform social activities proper to their age, going out to parties with friends, consuming alcohol, sightseeing or going overseas.

*One loses his youth ... the phase at which people go out to drink or party ... and I see my cousins, many of them are in the age that I started HD and they do different things, which I could not do ... it is a phase that can not be wasted ... young people want to go sightseeing ... to enjoy the world… (VG)*

At the same time, they have difficulty to establish social relationships with people of the opposite sex. They feel that, because they are under this condition, no one pays attention to them and that they are not attractive enough to establish relationships that go beyond a simple friendship. This is why there are feelings of frustration, dissatisfaction and rejection to the disease and the treatment itself, leading them to states of depression due to the situation they face.

*Sometimes I thought, “I am on dialysis to get a woman”, but it is hard winning over a woman who looks at you like that, besides, they are complicated, they use to say: “This sick guy ...“I felt traumatized ... depressed and I used to think “I so young and sick like this” I thought about many things. (JIB)*

In this same sense, the disease is shown to the others through the arteriovenous fistula, and they feel as if they were “from another world”, considering themselves as objects of the curiosity of others and experiencing feelings of shame, which are accentuated by the fact that they are young. 

*The fistula seems like something from another world... it seems bizarre here in this world ... because it arises people’s curiosity... mainly when you are young... there are ladies who do not care about what people say ... but a young man cares about it, that people look at it. ...even if they are not looking at it, you get it into your head...that people are paying attention to it. (VG)*

Being young causes the disease to be perceived as a factor that affects the development of social activities and the establishment of love relationships, besides feeling observed by having an arteriovenous fistula, which may be a way of showing the presence of a disease.

Relationality, aspects such as support, feeling stuck and Terminal Chronic Renal Failure were identified.

Support, there are two sub-themes within support, which are perceived in the disease, the support from friends and family support.

The support from friends. It occurs in different ways: at the economic level, providing housing, helping with food, collaborating at work; making them feel supported and that they can trust these friends. They share news related to the transplant with these persons and request their collaboration to solve the different situations that may arise. Thus, they transform their companionship into a way of understanding that “life is not over”, that they are still alive and, therefore, they must move forward in life, accompanied not only by the disease, but also by people who are there for them.

*I called some friends ... I told them “I will probably undergo the transplant”. I took some money and went to the airport, I traveled to Bogotá to undergo the transplant ... When I came to live with my friend ... his wife ... she used to cook the food without salt, I took out my food first and then she added salt for everyone, they used to take care of my diet. (EYC)*

*My roommates arrived, a blessing, the companions of admitted patients and they gave me a lesson about life, at a given moment they asked: What happened? Why? How was the surgery? because they always ask that, then they started talking about work, what happened at work, I kept looking at them and said “Life is not over, I’m alive, with this disease, but still alive”. (MCV)*

Family support. It is crucial to feel accompanied and protected, with the perception of being cared for. The intervention of the family at this stage gives them an impression that the problems diminish, helps them cope with the disease and facilitates the adaptation process. They recognize the participation of their family in the care of their diet, their economic and emotional support, as well as their participation in the treatment. In this way, they realize that the disease is not a process that is lived alone, but it involves all family members, making them indispensable pillars for the person to overcome the disease.

*The disease belongs to the two, three, four of us, it is not only mine; it also belongs to my husband and my children. The young partner wants the disease to be experienced only by his wife, but the affected person needs support from others to overcome the disease, it is vital having the family on their side. If you do not have family support, you have a relapse, and it happens quickly; if the husband does no support her wife, she will not accept her disease, she will not accept living with the disease ... and then Goodbye... this is the main cause, not accepting the disease. (MEA)*

Being stuck. The disease leads them to feel stuck, because they have difficulties to travel, go to work and attend leisure activities. The feeling of being stuck is described in their statements, they are excluded from many activities because the disease limits the possibility of carrying them out or they have to be postponed, and the fear they experience by the possibility of making a trip or by the inability to work because they feel stuck becomes evident.

*One feels stuck ... I had the opportunity to go abroad because I have a daughter who lives in another country, but I do not want to travel now, I’m afraid that I will get sick there ... I am afraid of having an urinary tract infection or something like that. (AS)*

*I had to look for a job that did not require much physical effort because I underwent a lung surgery ... to run ... I become breathless, I am not able to make much physical effort and the dust is bad for me because of the dialysis. (JIB)*

Terminal Chronic Renal Failure. In the interviews, the participants express the terror they experience when they hear the words terminal chronic, in reference to the disease in the stage in which they are, since they have the feeling of approaching death. Their statements reveal the distress they suffer when they hear words that have a frightening meaning for them. 

*It causes terror because when they say “there’s nothing left to do”, the word terminal means “it is not working anymore”, “you are going to die”, people have an impression that they say terminal, because: AIDS, cancer and brain death are terminal illnesses, then, the affected aspect is the psychological one. (EP)*

Spatiality, the following aspects were identified: changes in life, sadness and depression.

Changes in life - “a splash of cold water”. The disease leads to changes in their lives, making them to abandon their plans, adjust their lifestyle, adapt to a new situation, assume a new treatment and make decisions. They feel as life was over and they must awake to a hard reality, experiencing it as a surprise. In many cases, their lives suffer a heavy blow because they represent the economic support for their family, and all this leads them to rethink their situation, causing a role change, which has an impact on their lives in the economic, social and affective contexts.

*It is very hard ... it’s like when one is awake and someone else throws a bucket of cold water on him. What happened? Where from do I start? Where do I go? What do I do? What am I going to do with my life? Should I go, stop or stay? (EP)*

*Having to change my lifestyle, I was one of those who spent the night reading, I spent the night in a conference, a play, I had plans, to do a master’s, to work in the area of education, to continue in literary history, and in this fateful moment the person thinks: “I’ve already died”, I have a serious illness. (MCV)*

Sadness and depression. The disease affects their emotional condition, and they refer moments ranging from not knowing what to do to suicidal thoughts. They have feelings of dispair and sadness, which sometimes lead them to think about committing suicide. However, through the perceived support, reflection and medication, they manage to overcome these feelings and move on, accepting the disease. Loneliness and depression are factors that worse feelings of helplessness and the desire to end up their lives in order to end the suffering they go through.

*It was hard in the first 15 days ... I tried to commit suicide, and that story “it only happens to others”, to want to die, it’s true ... I thought: What now? When I started to think about it, I decided to commit suicide. I opened the window on the second floor and was about to jump, but when I opened the window, I realized: What am I doing? ... Forgive me, God, no, and then I realized that I was wrong, I was depressed, mainly because I was alone”. (MCV)*

Corporeality, the disease becomes visible: body deterioration and changes in sex life.

Body deterioration. The disease causes body deterioration, weight loss, changes the color of your skin, and abdominal bloating, which makes them insecure and fearful. They feel ashamed of their body, which prevents them from establishing relationships with others. The disease becomes evident in all its magnitude, since it converts the body into an instrument that makes it appear with all its intensity, consolidating its presence in their lives.

*“I was embarrassed when they looked at me ... they used to ask me ... What´s wrong with you? ... I was skinny and with a big belly, I was ashamed ... I had been shy all my life ... I used to think, but why do they keep asking so much? ... I did not feel like going out ... I preferred to stay home”. (MMV)*

*I have become skinny, brown, dark, because the body changes physically and is terrible, physically ... one feels ugly ... one feels different from others, from normal people ... then self-esteem decreases. (VG)*

Changes in sex life. The disease affects the sex life and, feeling sick and being in treatment are factors that cause them to have difficulties to maintain an active sex life. The sex life changes, it simply reduces and they experience fear of not “functioning”, associating it with uremia, drugs, comorbidities or the presence of the peritoneal catheter, which causes them distress and despair, in adittion to the aggravation of not being able to express the situation or seek help to solve it.

*Regarding the experience of having sex, one remains discouraged, the disease and many medications affect the erection... then one tries to lead the marriage as good as possible, but it is not the same anymore... I know friends who have problems in that sense, they do not express it because these are intimate things, and these are manifested during conversations, when there are meetings. ... let’s consider that then these are expressed. (AS)*

*The sexual rhythm has changed, it has been reduced simply to zero, and a concern arises: Do I look good enough for her? (MCV)*

Materiality, the aspects related to the effects on the economic status arise. 

Effects on the economic status. The statements allow perceiving the economic impact of the disease, as having to leave work or being retired for disability decreases their income. In adittion, the increasing costs of travel and the need to assume the costs of treatment lead them to perceive the disease as a difficult situation to overcome.

*Leaving my job cost me a lot, I was working, having to leave my job was the hardest part for me, I should have been earning a better salary, and now I have a low income ... then it hurts, I do not earn as much as I should ... it has been the highest cost due to the disease, I had to become a pensioner. (MCV)*

*The economic aspect is affected, I had to leave my job, I did not feel able to work, I felt tired ... I used to have physical disposition, but I did not feel in shape to work well anymore. (AS)*

## Discussion

Temporality, it is not the time of the clock or chronological time, but the time in which the phenomenon is experienced^13^, the past is the “pre”, the future is “what is to come” and the present is the “now”^14^. Therefore, the presence of the disease is recognized as something unexpected, but whose progression occurs over time, abstract, but that can be decisive in breaking the fragile line between health-disease. 

The abrupt appearance of the disease coincides with the results of studies that describe its presence as a clash between the past and the present, making it difficult to understand how it progressed so much. The diagnosis and treatment of the disease are perceived as sudden, overwhelming, and unknown^17^, and its discovery is belated because its symptoms are not perceived, which prevents its detection in a timely manner. Therefore, it is recognized the importance of promotion and prevention programmes that contribute to early detection and treatment of the disease in order to delay its progression^18^. 

Being present and not seeing it, the symptoms appear but they are not identified due to lack of knowledge. Anemia, proteinuria and edema occur in Chronic Kidney Disease, but they go unnoticed and are not evident, causing them to ignore their disease^19^. Thus, sick people would benefit from timely interventions of interdisciplinary nephrological teams that promote education and care strategies, aiming at minimizing the impact of the disease^20^, as the lack of knowledge contributes to the initiation of dialysis on an urgent basis, causing an increase in the morbidity and mortality rates in these people^21^.

Young people have difficulty accepting changes in their self-image, and they feel insecure about this situation. It has been described how this affects their social interaction, mainly because of the presence of catheters, arteriovenous fistula and the impossibility of carrying out activities of daily life, considered as stressors for them^22^. Besides, they also suffer from loneliness, isolation and loss of self-esteem associated with the presence of the disease and treatment^23^.

Research results describe the importance of family and friends in the care of the disease, which helps them to carry on with their lives^21^, as well as the perception of the negative impact that the disease has on their family due to the treatment^24^. 

The treatment, perceived as the central and essential axis of the activities of daily life, awakens in them feelings of dispair and anguish, which make them feel stuck in their own world^25^. They lose self-sufficiency, becoming dependent on others and treatment, and these restrictions, distress and limitations are perceived as “loss of freedom”, which restricts their social activities^26^.

As for changes in life, sadness and depression, several studies show how people with Chronic Kidney Disease, especially young people, experience perplexity and denial in relation to their condition, leading them to develop negative thoughts about their lives^27^, and to express surprise, anger, indignation, isolation and depression due to the fact of having the disease^28^. Other studies show how the disease makes them unable to carry out their activities, dietary and fluid restrictions, as well as restrictions on their lifestyle, which represent factors that affect the balance between illness and normality, making them understand that their lives have changed^11^.

Regarding the body deterioration, there are studies describing the existence of an interaction between the ill body, the treatment and the work, which leads to changes in the abilities and needs of the “chronically ill body”^29^. This deterioration exerts an influence on the lives of the patients and leads them to develop dependence, self-pity, depression and other alterations that produce an overload and a worsening in their quality of life^30^. 

Chronic Kidney Disease and hemodialysis, mainly, significantly affect people’s sex life, increasing the impact that the disease has on those who suffer from it^7^. The descriptions show how men and women experience the effects of the disease, and the loss of sexual interest associated with the disease and treatment, which is accepted with resignation^31^. In this sense, the presence of a catheter is perceived as a threat to marital relationships, becoming an obstacle in sexual relations^32^.

The findings of this review show that the symptoms of the disease have a negative influence on working life^31^. The disease itself produces an adverse economic impact, taking into account the time spent on treatment and the additional costs that patients must assume for doing so. Keeping their jobs allows them to meet their needs and feel fulfilled as a person, so they feel afraid of having to leave their job, because that means depending on their family or stop being the economic support for them^26^. Changing their working life brings them financial difficulties and, in many cases, they become disable and must be away from work, which represented a mechanism for them to feel normal in their lives^18^.

## Conclusions

Chronic Kidney Disease appears unexpectedly, abruptly; it manifests over time and they recognize that there were signs that were underestimated because they could maintain their daily life. Young people have their social interactions impaired and are marked by a social stigma due to changes in their body image. The disease may cause body deterioration, which changes the patient’s perception in relation to how this sick body is experienced. 

The family and friend support is a way to move forward in life, which allows them to cope with the dependence caused by the disease and treatment.

There are changes that lead them to rethink their lives, feelings of depression, distress and fear appear; suicidal ideas can be associated with the loss of control in their lives; having support, affection and reflecting on this situation are important mechanisms to assume the disease with responsibility. The disease and treatment have effects on their economic situation; abandonment of work, dependence and increased economic burden leads them to lose the “normality” that they wish to experience in order to face the disease.

This study represents an important tool for the development of self-care models articulated with public health policies aimed at caring for people with Chronic Kidney Disease. It is based on the experience of people, on the subjectivity and meanings that these people build, moving away from the positivist paradigm centered on the disease, to focus on the care for human beings, authors and participants of their own reality. 

## Study limitations

This study shows interesting aspects for the understanding of the experience of people living with Chronic Kidney Disease; its limitation is related to the difficulty to generalize its results, although they may shed light on aspects that can be configured as similar in people living with Chronic Kidney Disease.
